# Atypical MAPKs in cancer

**DOI:** 10.1111/febs.17283

**Published:** 2024-09-30

**Authors:** Katrin Dahm, Parthiban Vijayarangakannan, Hans‐Peter Wollscheid, Hansjörg Schild, Krishnaraj Rajalingam

**Affiliations:** ^1^ Cell Biology Unit University Medical Center Mainz, JGU‐Mainz Germany; ^2^ Svastia Genetics, Future Business Centre Cambridge UK; ^3^ Institute of Immunology University Medical Center Mainz, JGU‐Mainz Germany

**Keywords:** cancer therapy, ERK3, ERK4, ERK7/8, MAPK4, MAPK6, MAPK15, Nemo‐like kinase

## Abstract

Impaired kinase signalling leads to various diseases, including cancer. At the same time, kinases make up the majority of the druggable genome and targeting kinase activity has proven to be a successful first‐line therapy for many cancers. Among the best‐studied kinases are the mitogen‐activated protein kinases (MAPKs), which regulate cell proliferation, differentiation, motility, and survival. However, the MAPK family also contains the atypical members ERK3 (MAPK6), ERK4 (MAPK4), ERK7/ERK8 (MAPK15), and NLK that are functionally and structurally different from their conventional family members and have long been neglected. Nevertheless, in recent years, important roles in carcinogenesis, actin cytoskeleton regulation and the immune system have been discovered, underlining the physiological importance of atypical MAPKs and the need to better understand their functions. This review highlights the distinctive features of the atypical MAPKs and summarizes the evidence on their regulation, physiological roles, and potential targeting strategies for cancer therapies.

## Graphical abstract

Mitogen‐activated protein kinases (MAPKs) regulate various cellular processes, including proliferation, differentiation, and motility. Alterations in these signalling pathways can lead to various diseases, including cancer. The atypical MAPKs ERK3 (MAPK6), ERK4 (MAPK4), ERK7/8 (MAPK15) and NLK have long been neglected in kinomic studies. This review highlights their unique characteristics and important physiological roles, as well as their potential for targeting strategies in cancer therapy. Created with BioRender.com.

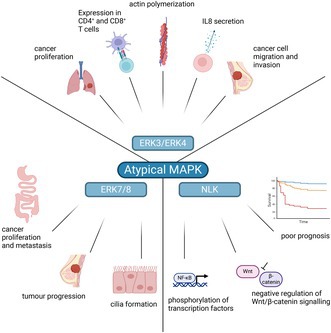

**Abbreviations**
AR: androgen receptor
ARP2/3: actin‐related protein
BCR‐Abl: breakpoint cluster region Abelson
BRCA: breast invasive carcinoma
C/EBP: CCAAT/enhancer‐binding protein
C34: conserved in ERK3 and ERK4
CESC: cervical squamous cell carcinoma
CML: chronic myeloid leukaemia
COAD: colon adenocarcinoma
DGK: diacylglycerol kinase
ERK: extracellular signal‐regulated kinases
ERα: estrogen receptor alpha
FBXW7: F‐box and WD repeat domain‐containing 7
FOXO1: forkhead box protein O1
FOXP3: forkhead box protein P3
GRα: glucocorticoid receptor alpha
HNSC: head and neck squamous cell carcinoma
HPV: human papillomavirus
IL‐8: interleukin‐8
JNK: c‐Jun amino (N)‐terminal kinase
LUSC: lung squamous cell carcinoma
MAPK: mitogen‐activated protein kinase
MAPKAPK5, MK5: MAPK‐activated protein kinase 5
NFκB: nuclear factor kappa B
NLK: Nemo‐like kinase
NSCLC: non‐small‐cell lung cancer
PAAD: pancreatic adenocarcinoma
PAK1: group I p21‐activated kinase
PCNA: proliferating cell nuclear antigen
PROTAC: proteolysis targeting chimeras
RAC1: Ras‐related C3 botulinum toxin substrate 1
SRC‐3: steroid receptor coactivator 3
STUB1: STIP1 homology and U box‐containing protein 1
TAK1: TGF‐beta activated kinase 1
TGFβ: transforming growth factor beta
THCA: thyroid carcinoma
TNBC: triple‐negative breast cancer

## Introduction

Cells routinely transduce extracellular chemical and physical signals into various adaptive intracellular responses. Mitogen‐activated protein (MAP) kinase cascades are highly conserved signalling modules that play an important part in this process. The classical MAP kinase cascade is organized into three sequentially acting kinases [1]: a MAP kinase kinase kinase (MAPKKK) activates a MAP kinase kinase (MAPKK or MEK), which finally activates the effector MAP kinase that phosphorylates in turn a wide variety of substrates, including transcription factors, protein kinases, cytoskeleton‐associated proteins, and others, present in various subcellular compartments. As the name suggests, the first MAPKs discovered played an important role in regulating proliferation and mitosis by responding to extracellular mitogens [2]. However, it was later discovered that MAP kinases including the atypical MAPKs, regulate a wide range of cellular functions in response to various stimuli.

Efficient and accurate signalling through MAPK modules relies on scaffold proteins and docking interactions achieved through the common docking (CD) domain of MAPKs [3, 4, 5]. Within the same cell, several distinct MAPK modules may control diverse cellular processes, such as cell proliferation, differentiation, survival, and immune responses. The mammalian 2family of MAPKs consists of 14 members (10 conventional and 4 atypical MAPKs) that define seven distinct MAP kinase pathways. The conventional MAPKs comprise extracellular signal‐regulated kinases (ERK) 1/2, c‐Jun amino (N)‐terminal kinase (JNK) 1/2/3, p38α/β/γ/δ, and ERK5, which are well studied for their physiological roles, regulation, and substrate specificity [6, 7, 8, 9, 10]. In contrast, the atypical MAPKs ERK3 (MAPK6), ERK4 (MAPK4), ERK7/ERK8 (MAPK15) and Nemo‐like kinase (NLK), which differ both functionally and structurally from the classical MAPKs, have long been overlooked by researchers. Like their conventional family members, atypical MAPKs are protein Ser/Thr kinases, however, they are neither organized into classical three‐tiered kinase cascades nor do they necessarily possess the characteristic Thr‐X‐Tyr motif in their activation loop. Instead of two phosphorylation sites, their activation motifs often have only one phospho‐acceptor site, such as the SEG motif in ERK3 and ERK4. In addition, atypical MAPKs which have been discovered based on their sequence homology to conventional MAPKs are characterized by unique structures of their C‐terminal tails [2].

Recent discoveries have unveiled exciting cellular functions and critical (patho‐)physiological roles of several members of this family branch, often involving kinase‐independent mechanisms of action. In the following, we highlight the structural features and peculiarities of atypical MAPKs and discuss how they enable their physiological functions but also contribute to disease and therapy resistance, particularly related to cancer.

## Structural features of atypical MAPKs

**Fig. 1:**
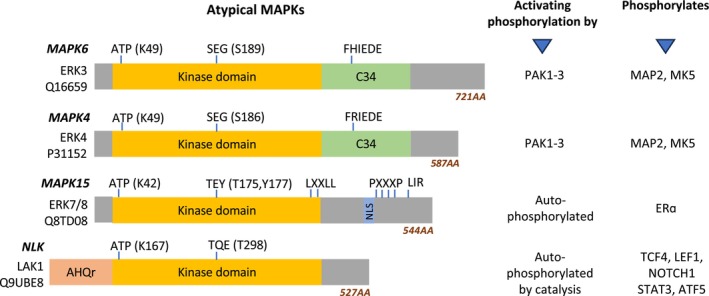
Atypical MAPK domains, ATP‐binding and phosphorylation sites, and functional motifs. Atypical MAP kinases' gene names and UniProt IDs are listed, along with the protein kinase and other domains. The SEG, TEY, and TQE motifs of ERK3/4, ERK7/8 and NLK respectively contain the activating phosphorylation sites. ERK3 and ERK4 contain the C34 (shortened form of ‘Conserved in ERK3 and ERK4’) domains that harbour the FHIEDE or FRIEDE motif respectively in the C‐terminal region. A conserved ATP‐binding site (Lysine, K) is present in each member in their respective kinase domains. ERK7/8 contains multiple PXXXP motifs in the C‐terminal region that regulate chromatin‐binding and interaction with PCNA, as well as a Nuclear Localization Sequence (NLS) enabling its translocation into the nucleus upon various stimulations. ERK7/8 also contains two C‐terminal LXXLL motives and a LC3‐interacting region (LIR) that enable binding with ERRα and interactions with LC3 respectively. The diagram was drawn using coordinates and information retrieved from the Uniprot database.

**Fig. 2:**
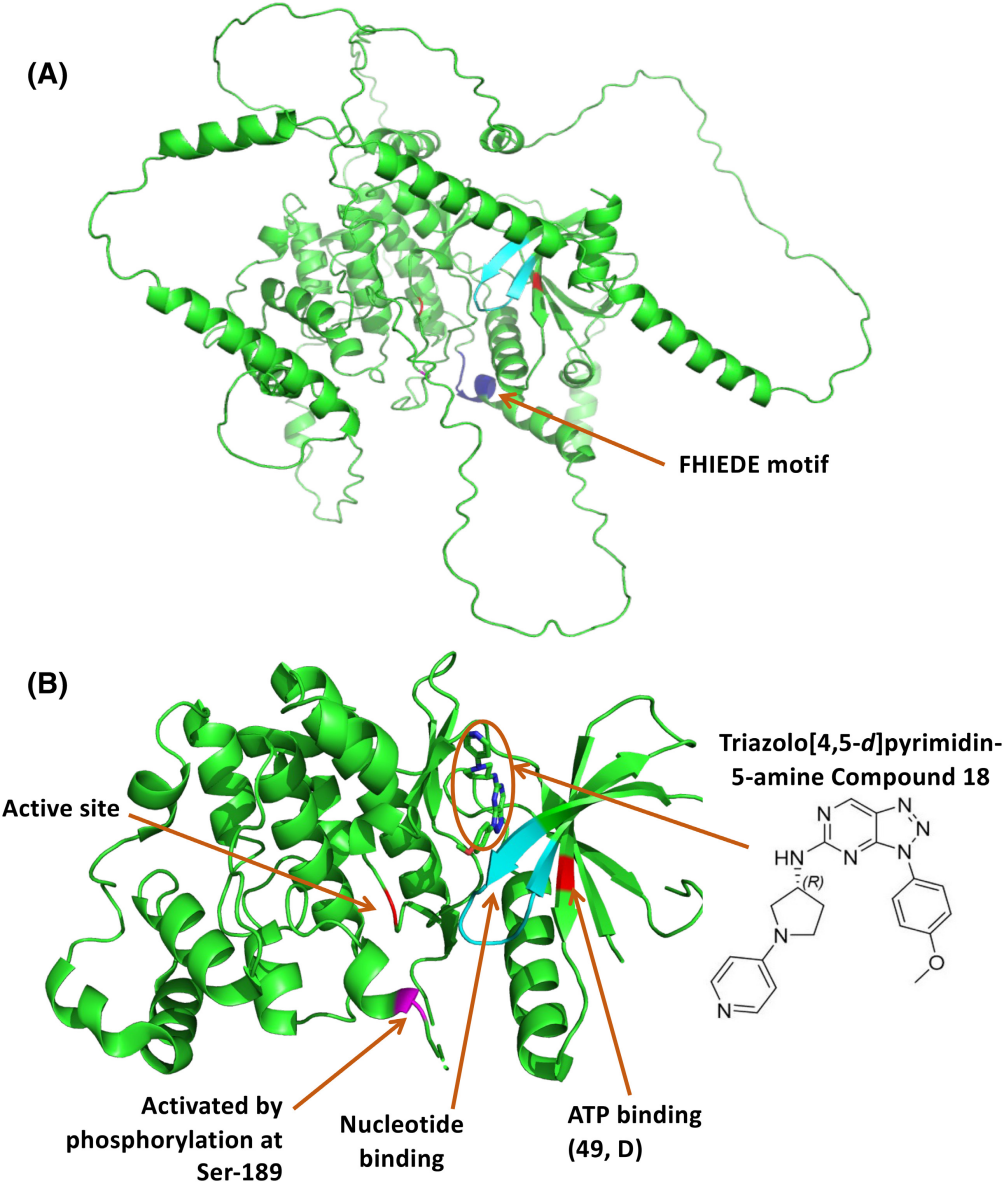
ERK3 structure, binding sites and druggability. (A) ERK3 protein structure, complete with the loops and tails, as predicted by Alphafold (Alphafold ID: AF‐Q16659‐F1, Uniprot: Q16659). The FHIEDE motif (332–337) that interacts with MK5 is highlighted and annotated. (B) ERK3 X‐ray structure in the PDB (ID: 6YKY), docked with Triazolo[4,5‐*d*]pyrimidin‐5‐amine (Compound 18) published by Gradler *et al*. [45], showing multiple functional domains including the key phosphorylation site (189, S), ATP‐binding site (49, D) and the nucleotide‐binding site (26–34, LGCGGNGLV). Elkhadragy *et al*. [44] reported the C‐terminal tail's involvement in cell invasion and motility. Given that the experimentally validated ERK3 PDB structures exclude the C‐terminal region, the structure predicted by Alphafold currently is the only interpretation of secondary structural elements in this region. It is noteworthy that while certain secondary structure elements in this region, such as α‐helices, have a high pLDDT score (above 50), the C‐terminal region's pLDDT score falls below 50. This suggests that the C‐terminal is likely to be flexible and unstructured, potentially interacting within the grooves of ERK3's binding or signalling partners, including Septin 7. Furthermore, the Alphafold structure allows for interpretation of the spatial relationship of the FHIEDE motif with adjacent side chains or other structural elements. In addition, Alphafold 3 [111] could be used to predict the binding pockets of molecules that target ERK3. Due to low Alphafold pLDDT scores in the disordered regions, any structural analysis should involve caution to avoid misinterpretation. Structures in PDB format were downloaded from the Alphafold and PDB databases for (A) and (B) respectively. pymol v2.5.0 (Schroedinger, Inc., NYC, New York, NY, USA) was used to visualize and annotate the functional domains/motifs using the information from the Uniprot database.

**Fig. 3:**
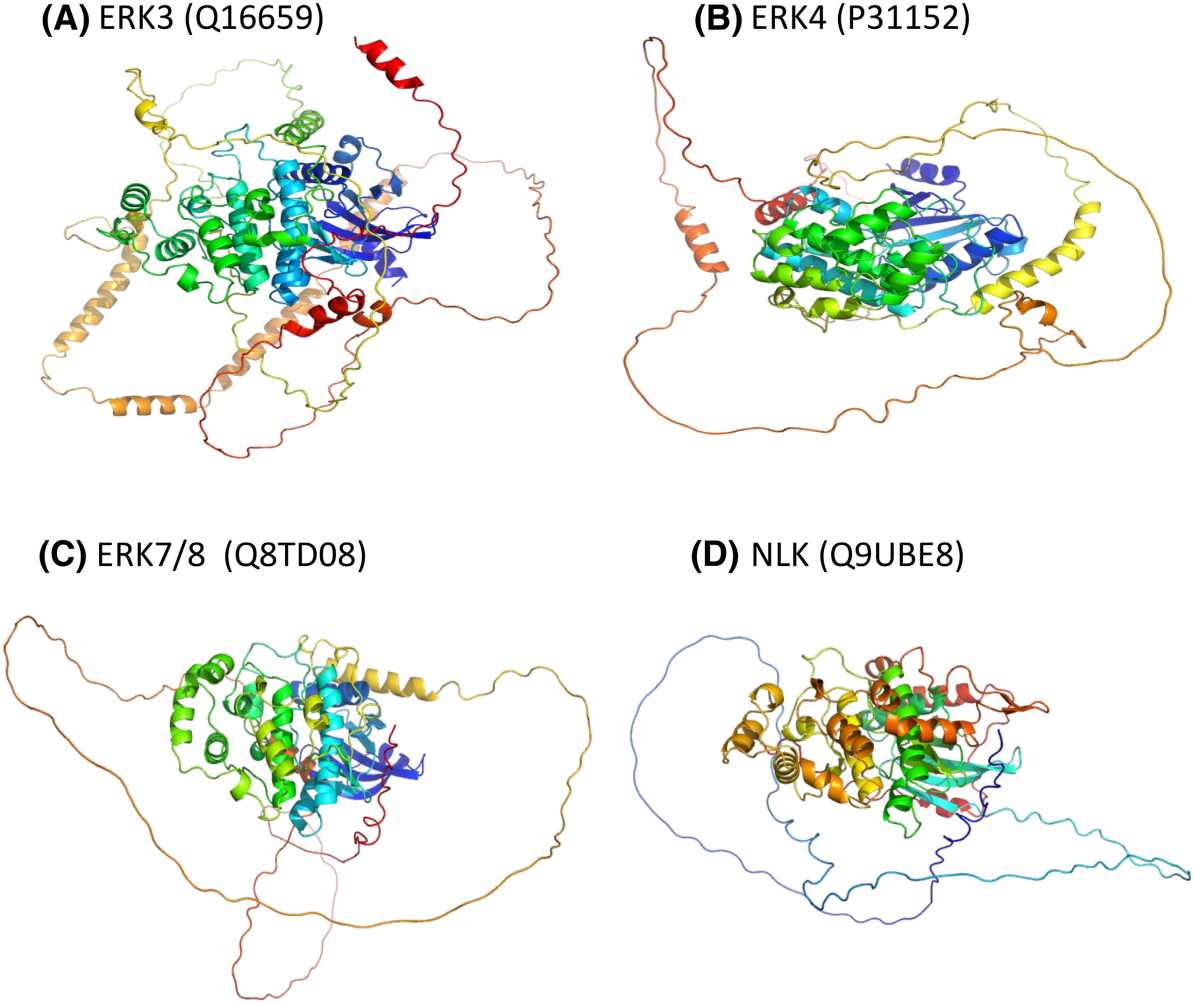
Structures of atypical MAPKs as predicted by Alphafold. (A) ERK3, (B) ERK4, (C) ERK7/8, and (D) NLK. The structures were inherently refined by the secondary structural features of MAPK crystal structure of ERK3 (PDB ID: 6YKY). The atypical MAPKs are characterized by the C‐terminal, disordered, tail‐like regions and the presence of a single residue in the activation loop that can be phosphorylated. The experimental PDB structures lack the C‐terminal regions whose functional significance is still under extensive research. The 3D structure images were downloaded directly from the Alphafold database.

Similar to classical MAPKs, all atypical MAPKs contain a Ser/Thr kinase domain with a conserved ATP‐binding site and the motifs SEG (ERK3/4), TEY (ERK7/8) or TQE (NLK), which contain the activating phosphorylation site(s) [11] (Figs 1, 2, 3). The kinase domains are N‐ and C‐terminally flanked by regions of different lengths and functions. ERK3 and ERK4 contain the C34 (Conserved in ERK3 and ERK4) domains that harbour the FHIEDE or FRIEDE motif, respectively, in the C‐terminal region (Figs 1 and 2A). These motifs enable the interaction with their substrates and the ubiquitin E3‐ligase F‐box and WD repeat domain‐containing 7 (FBXW7), which mediates ERK3 ubiquitination and degradation [12].

ERK7/8 (MAPK15) contains multiple PXXXP motifs in the C‐terminal region (Fig. 1) that regulate chromatin‐binding and interaction with PCNA, as well as a Nuclear Localization Sequence (NLS) enabling its translocation into the nucleus upon various stimulations. NLK contains an Ala‐His‐Glu‐rich region (AHQr) at the N‐terminal end (Fig. 1) that binds to ZIPK (Zipper‐interacting protein kinase), halting or reducing the repression of Wnt/β‐catenin signalling [13].

## ERK3 (MAPK6)

ERK3, the best studied atypical MAPK, is ubiquitously expressed in all tissues. Known physiological substrates include the protein kinase MK5 (MAPK‐activated protein kinase 5, MAPKAPK5) [14, 15, 16], microtubule‐associated protein (MAP2) [17, 18], tyrosyl DNA phosphodiesterase 2 (TDP2) [19], steroid receptor coactivator 3 (SRC‐3) [20], septin‐7 [21], supervillin (SVIL) [22], diacylglycerol kinase DGKζ [23], ARP3 [24], and AKT [25]. Of these, MK5 is the most commonly used substrate to determine ERK3 activity, *in vitro* and *in vivo*. MK5, a serine/threonine kinase with known functions in actin remodelling and cell migration forms a complex with ERK3 via the FHIEDE motif of ERK3, leading to MK5 phosphorylation and subsequent activation at Thr‐182 [26]. In resting cells, ERK3 is constitutively phosphorylated at Ser‐189 within the SEG motif (Fig. 2B), but due to N‐terminal ubiquitination and proteasomal degradation, ERK3 is an unstable protein with a half‐life of only 30 min [27, 28]. De‐ubiquitination by USP20 was shown to stabilize ERK3 resulting in actin cytoskeleton remodelling and enhanced cell migration [29]. Thus, ERK3 levels likely reflect its activity. In addition, group I p21‐activated kinases (PAKs) involved in actin cytoskeleton regulation mediate Ser‐189 phosphorylation in response to RAC1 activation [30], and Ser‐189 phosphorylation was also increased upon KRAS overexpression [31]. Despite recent progress, the regulation and physiological roles of ERK3 remain poorly understood, hinting towards partly contradictory, cell type‐ and tissue‐specific functions in cell proliferation and migration [20, 31, 32, 33, 34, 35], chemoresistance [19], differentiation [36, 37], insulin secretion [17], and lipolysis [38]. For example, it was shown that RhoGTPase activation, which is decreased upon ERK3‐depletion, was partially rescued upon EGF stimulation in primary cell types (HMECs) but not in cancer‐derived cells (MDA‐MB231) [24]. Similarly, in the context of LPS stimulation, ERK3 was described to be destabilized in primary cells (HCPECs) but shows increased stability in tumourigenic cells (HT‐29) [36].

### ERK3 in cancer

**Fig. 4:**
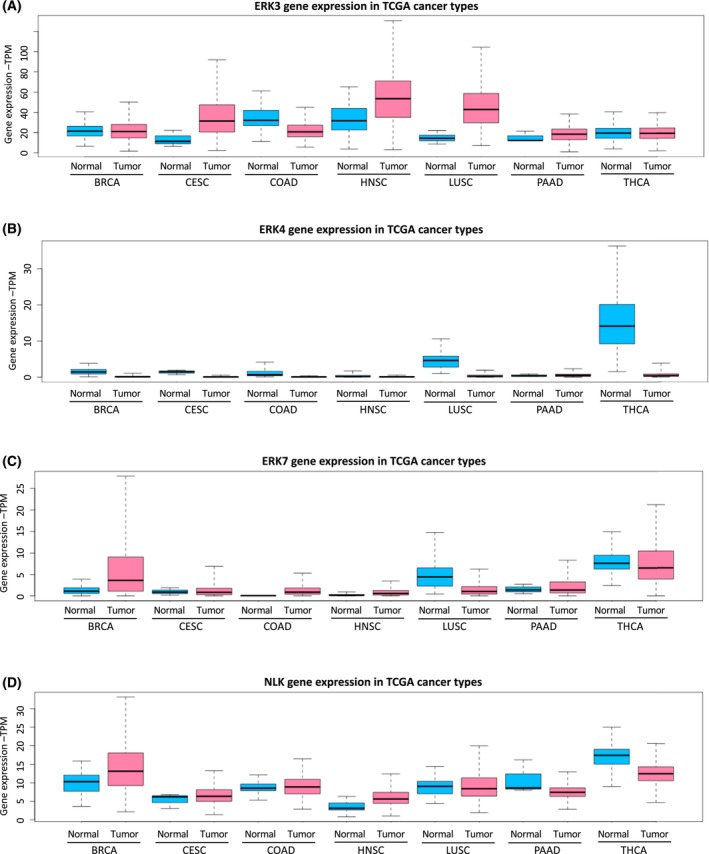
Gene expression of atypical MAPKs in various tumour and normal samples: (A) ERK3, (B) ERK4, (C) ERK7/8 and (D) NLK. By analysing the TCGA RNA‐Seq data (normalized RNA counts), it is possible to identify the types of cancer for which certain atypical MAPKs could be a promising drug target. Normalized gene expression counts were obtained from the UALCAN portal. UALCAN utilizes the ‘Primary Solid Tumour’ and ‘Solid Tissue Normal’ gene counts in TCGA for each cancer [112]. Normal and tumour samples have the suffixes ‐N and ‐T respectively. Tumour study abbreviations are available from the TCGA Genomic Data Commons data portal (https://gdc.cancer.gov/resources‐tcga‐users/tcga‐code‐tables/tcga‐study‐abbreviations) [113]. BRCA, breast invasive carcinoma; CESC, cervical squamous cell carcinoma; COAD, colon adenocarcinoma; HNSC, head and neck squamous cell carcinoma; LUSC, lung squamous cell carcinoma; PAAD, pancreatic adenocarcinoma; THCA, thyroid carcinoma.

The expression of ERK3 is known to be upregulated in several human cancers: Non‐small‐cell lung carcinoma (NSCLC) [31, 34, 35, 39], breast cancer [40], gastric cancer [41] and melanoma [42, 43]. Additionally, our analysis of TCGA derived patient data showed particularly high transcription levels of ERK3 in squamous cell carcinomas CESC, HNSC and LUSC (Fig. 4A). Notably, LUSC is one of the most common types of NSCLC, next to large‐cell carcinomas and adenocarcinomas [31].

In NSCLC, oncogenic KRAS was found to stabilize the ERK3 protein and enhance phosphorylation at Ser‐189 across all tumour stages. ERK3 depletion markedly decreased oncogenic growth of KRAS‐mediated anchorage‐independent growth of NSCLC cells *in vitro* and in mice [31]. In addition, the effect of ERK3 on NSCLC cell growth was shown to be kinase‐dependent. The reintroduction of ERK3 wild type into ERK3‐depleted immortalized lung epithelial (SALEB‐KRAS) cells rescued the reduced growth, whereas the reintroduction of an ERK3 kinase dead mutant did not show this effect. Thus, ERK3 as a protein or its kinase activity could be a potential therapeutic target in the treatment of KRAS‐driven tumours. TCGA‐derived patient data show that ERK3 is upregulated in tumours regardless of the KRAS (or EGFR) mutation status suggesting complex relationships and multi‐layered roles for ERK3. As of now, there are only a few known regulators of ERK3, and the mechanisms by which KRAS upregulates or stabilizes ERK3 remain largely elusive. ERK3 was also found to promote migration and invasiveness of both KRAS‐mutated and KRAS‐wild‐type NSCLC cells by phosphorylating steroid receptor coactivator 3 (SRC‐3), a bona fide oncogene [20]. Another target of ERK3 in NSCLC cell lines is the phosphodiesterase TDP2, which was activated when phosphorylated by ERK3. This upregulated TDP2‐mediated DNA damage response and desensitized lung cancer cells to Top2 inhibitor‐induced growth inhibition [19].

Recently, ERK3 has been implicated in the regulation of the epithelial secretome in response to lipopolysaccharide (LPS) [36]. Interestingly, the kinase activity of ERK3 is not required in this process. ERK3 was rather found to physically interact with c‐Jun to regulate the activity of the transcription factor AP‐1 and thus mRNA levels of several chemotactic factors including CXCL8/IL‐8, a prime modulator of epithelial immune responses. ERK3‐mediated IL‐8 secretion was critical for the chemotaxis of leukocytes to the epithelium *in vitro* and *in vivo* [36]. Remarkably, LPS stimulation has different effects on the ERK3 protein levels in oncogenic and primary epithelial cells: whereas ERK3 protein levels in primary (HCPEC) cells decreases over time, they remain at a high level in tumourigenic cells (HT‐29). Indeed, in addition to a role in epithelial immune defence, this ERK3‐IL‐8 axis could also be important for carcinogenesis. It has been shown that breast cancer cells (MDA‐MB231), whose migration and metastatic potential is enhanced by IL‐8, have a significantly reduced potential to form metastases in mice when ERK3 was depleted [36].

### ERK3 in cancer cell migration

Recent findings suggest that ERK3 can modulate cell motility in a multitude of ways to promote cancer cell migration and invasion. ERK3's C34 domain binds two downstream modulators of cancer cell motility: the cytoskeletal protein septin 7 [21, 44] and the diacylglycerol kinase DGKζ [23], allowing their phosphorylation by ERK3. In addition, ERK3 was found to directly interact with and regulate two key molecules in actin cytoskeleton regulation: ARP3 and the RhoGTPase CDC42 [24]. *In vitro*, ERK3 acted both as a direct guanine nucleotide exchange factor (GEF) for CDC42 and as a nucleation‐promoting factor of ARP2/3‐dependent actin polymerization, and silencing of ERK3 in cells prevented both basal and EGF‐dependent CDC42 activation, filopodia formation and epithelial cell migration. Interestingly, although the ERK3 kinase activity was necessary for the formation of actin‐rich protrusions in mammalian cells (e.g. primary mammalian epithelial HMEC, or cancer‐derived MDA‐MB‐231 cells) and ERK3 directly bound to the ARP2/3 complex, phosphorylating ARP3 at Ser‐418, the kinase activity of ERK3 was dispensable for the F‐actin accumulation in HMEC cells. This suggests that ERK3's kinase activity may play a crucial role in bundling and/or branching rapidly polymerizing actin filaments, rather than in actin polymerization itself [24]. This example also shows how kinase‐dependent and ‐independent functions of atypical MAPKs can intertwine.

### ERK3 as anticancer drug target

Targeting ERK3 in cancer could involve the inhibition of its kinase domain, thereby modulating the phosphorylation of downstream effectors, and/or the C‐terminal region containing the C34 domain and the FHIEDE motif, thereby interfering with protein–protein interactions. Furthermore, inhibition of the kinase activity might lead to degradation of the protein as well. In any case, the specific activities and physiological functions of ERK3 and their implications for safety/toxicity upon ERK3 inhibition should be considered as it will affect substrates that may be essential for physiological functions, like its role in the regulation of actin dynamics upon growth factor stimulation or cell cycle regulation via supervillin phosphorylation during cytokinesis [22]. However, as still little is known about the physiological role of ERK3, it is currently difficult to predict the effects of its inhibition. Moreover, since ERK3 and ERK4 share high sequence homology, any small molecule inhibitor may lead to full or partial inhibition of both kinases.

A first series of recently developed ERK3 kinase inhibitors, which target the ATP‐binding pocket, exhibited excellent kinase selectivity and high potency to inhibit MK5 phosphorylation *in vitro* and in cells [45]. Stable protein structures with some of these compounds docked with the ERK3's PDB structure hinted towards comprehensive effects on MK5 phosphorylation and the auto‐phosphorylation of ERK3 and suggest a potential structural impact on the FHIEDE motif, i.e., substrate binding. Efficient inhibitors that specifically bind to the C‐terminal part and disrupt the C34 domain's binding with multiple partners, such as ZIPK or septin‐7 [23, 44, 46], are yet to be developed. However, the C‐terminal region of ERK3's protein structure is inherently unstable (Fig. 3A) (binding to its partners enhances stability), making conventional structure‐based approaches in inhibitor development challenging.

## ERK4 (MAPK4)

Due to similar genomic organization and high amino acid identity, ERK4 is considered as a paralog of ERK3 and the two kinases can form functional heterodimers [15]. Major differences are mainly present in the C‐terminal extensions of the proteins (Fig. 3A,B). Moreover, while ERK3 is ubiquitously expressed, ERK4 mRNA expression is restricted to the brain (highest expression), colon, eye, heart, kidney, lung, ovary, pancreas, placenta, prostate, and skin. Like ERK3, ERK4 is activated through phosphorylation by PAKs (PAK1–3), and MAP2 and MK5 are downstream targets. Both contain the FRIEDE motif (FHIEDE sequence in ERK3, Fig. 1), which facilitates the interaction with MK5. ERK4 and MK5 phosphorylate and activate each other iteratively. Binding to MK5 markedly increases the phosphorylation of ERK4 at Ser‐186, but this is independent of the kinase activity of MK5 [47]. Thus, the interaction between MK5 and ERK4 may increase the kinase activity of ERK4 and autophosphorylation [48] and/or prevents its dephosphorylation by phosphatases such as the MAP kinase phosphatase DUSP2 [49].

Genetic ablation of ERK4 had no effect on morphology or physiology of the mice; however, targeted inactivation may lead to depression‐associated behaviour in forced swimming test [50, 51]. ERK4 interacts with multiple proteins and pathways, including protein folding and processing, cilium assembly, HSP90 chaperone cycle, ERBB2 signalling, and others [52]. Direct key interactions from STRING [53] and IntAct databases [54] include PAK1–3 (P21 activated kinases 1–3), RAC1 (Ras‐related C3 botulinum toxin substrate 1), ELAC1 (Zinc phosphodiesterase ELAC protein 1), HSPB1, HSP90AA1/HSP90AB1 (heat shock protein/90 alpha/beta family members), IRAK1 (interleukin 1 Receptor‐Associated Kinase 1), ERC1 (ELKS/RAB6‐Interacting/CAST Family Member 1), PRKDC, and RAD23B amongst others. PRKDC is one of the most widely mutated proteins (7.82%) in lung/colon adenocarcinoma, breast invasive ductal adenocarcinoma, melanoma, endometrial adenocarcinoma, etc. [55]. RAD23B is implicated in breast cancer progression and its interaction with ERK4 may be of importance, either as a biomarker for predicting treatment response or as a potential target. The development of specific ERK4 inhibitors has not been in the focus of researchers so far, however, given its high similarity to ERK3 it can be expected that inhibitors targeting ERK3 will also—at least partially—inhibit ERK4.

## ERK7/ERK8 (MAPK15)

MAP K15 was identified first in rats (ERK7) and later in humans (ERK8) [56, 57]. ERK7 and 8 have an overall sequence identity of 69%, with 82% and 53% identity between their kinase domain and the C‐terminal regions, respectively. Despite differences observed in early studies, ERK7 (rat and mouse) and ERK8 (human) are true homologues, so the name for all three species has been changed to ERK7/8 (HGNC gene symbol: MAPK15) [58, 59].

The activating TEY motif of ERK8 contains two phosphorylation sites (T175 and Y177) [60], but an upstream activating kinase has not yet been discovered [59, 61]. Mutation of either Thr‐175 or Tyr‐177 to alanine or mutation of the ATP‐binding lysine (Lys‐42) results in loss of kinase activity of ERK8 although the other site remains phosphorylated [56, 60]. Ubiquitination was hypothesized to regulate ERK8 turnover [62]. The N‐terminal 20 amino acids of rat ERK7/8 are necessary and sufficient for proteasomal degradation; however, the enzymes involved and the ubiquitylation site(s) in MAPK15 are still unknown.

ERK8's physiological roles vary in different cell types [59] and are associated with its localization in various cellular compartments, including the cytoplasm [63, 64], cilium basal body [63], autophagosome [64], the Golgi apparatus [65], and the nucleus [66, 67, 68]. It can act as a proto‐oncogene or tumour suppressor, promote cell proliferation and transformation, stimulate autophagy, and regulate ciliogenesis, transcription, and protein secretion [59]. Moreover, Groehler and Lannigan [66] demonstrated ERK8, which is active in primary mammary cells but inactivated in breast cancer cell lines, interacts with Proliferating Cell Nuclear Antigen (PCNA), thereby preventing its degradation und supporting correct transfer of genetic information.

### Regulation of the nuclear receptor superfamily by ERK8

ERK8 was found to regulate transcription mediated by several members of the nuclear hormone receptor family, including estrogen receptor alpha (ERα) [69], androgen receptor (AR) [70], glucocorticoid receptor alpha (GRα) [70], and estrogen‐related receptor alpha (ERRα) [67]. ERα plays a critical role in breast and endometrial cancer, where loss of ERα has been shown to lead to aggressive tumours and poor clinical outcomes. ERK8 protein levels are decreased in breast cancer, and it was shown that this correlates with increased expression of ERα and tumour progression in breast cancer cell lines [69]. ERK8 enhances ubiquitination and proteasomal degradation of hormone‐bound ERα in a kinase‐dependent manner [69]. It is assumed that hormone binding leads to a conformational change in the ligand‐binding domain of ERα, which is subsequently ubiquitinated. Although the degradation of ERα was dependent on ERK8 kinase activity, phosphorylation of ERα did not play a role in the process. Instead, ERK8 may target a component of the ubiquitin machinery or an ERα‐interacting protein to facilitate ERα ubiquitination.

Rossi *et al*. [67] reported a direct interaction between ERRα and ERK8 via two LXXLL motifs (Fig. 1) in the C‐terminal domain of ERK8 *in vitro* using the yeast two‐hybrid assay and in breast cancer cells. ERRα is an orphan transcription factor that is constitutively active in the absence of ligands and strongly expressed in breast cancer. Its overexpression is associated with poor prognosis in triple‐negative breast cancer (TNBC). The interaction with ERK8 induces the translocation of ERRα to the cytoplasm and, consequently, the inhibition of its transcriptional activity.

### ERK8 in tumour progression

The proto‐oncogene c‐Jun is involved in tumour development and progression of various cancers [71]. As a transcription factor, c‐Jun is a part of the activator protein‐1 complex and is involved in several cellular activities, including proliferation, transformation, and apoptosis [72]. Its activity and stability depend on the phosphorylation of two serine residues, Ser‐63 and Ser‐73. MAPK15 has been shown to phosphorylate c‐Jun at these sites in colon cancer [73], osteosarcoma [74], and gastric cancer [75]. Knockdown of ERK8 blocked c‐Jun phosphorylation [73, 74, 75] and decreased metastasis in osteosarcoma *in vitro* and *in vivo* [74]. In gastric cancer, ERK8 knockdown resulted in cell cycle arrest and consequently reduced cell proliferation [75]. Furthermore, ERK8 was found to be overexpressed in carcinoma tissue of gastric cancer patients [75], as well as in metastasized osteosarcoma patients [74]. All three studies indicate that ERK8 promotes the development and proliferation of each cancer type and in addition promotes metastasis in osteosarcomas.

In chronic myeloid leukaemia (CML), ERK8 has been shown to play a role in the induction of autophagy by the oncogenic fusion protein BCR‐ABL1 [76]. BCR‐ABL1 is a constitutively active tyrosine kinase which activates various signalling pathways and which has been shown to be sufficient for leukaemia development [77]. This leads to altered cell function, including increased proliferation and decreased apoptosis. BCR‐ABL1 has been shown to interact with and activate ERK8 in CML cell lines, resulting in ERK8 re‐localizing BCR‐ABL1 to autophagic vesicles and inducing autophagy [76]. This appears to be the key process for BCR‐ABL1‐induced development of leukaemia, as well as for inhibition of apoptosis in leukaemia cells after drug treatment. Silencing ERK8 expression or using ERK8 inhibitors suppressed proliferation and transformation induced by BCR‐ABL1 *in vitro*. In addition, shRNA‐mediated knockdown of ERK8 lead to decreased tumour formation in mice. These results indicate an interesting approach in CML therapy and present ERK8 as a novel drug target.

### ERK8 and ciliogenesis

Cilia are microtubule‐based structures that mediate cellular motility, transmit environmental stimuli, and control various signalling pathways, including oncogenic Hedgehog signalling. Studies have shown that ERK8 is required for the formation of motile and primary cilia [78, 79, 80]. The underlying mechanisms are not yet clear, but ERK8, localized at the distal end of the basal body, appears to be responsible for the correct localization of ciliary proteins [78, 80]. Inhibition of ERK8 kinase activity disrupted ciliogenesis and inhibited Hedgehog signalling in medulloblastoma, one of the few cancers that forms cilia and in which Hedgehog signalling is often active in humans. Therefore, the inhibition of ERK8 lead to a reduction in the population of cancer stem cells and made the tumour less malignant [80].

## Nemo‐like kinase

Nemo‐like kinase is activated by autophosphorylation of Thr‐298 within its TQE motif and needs to homodimerize for intermolecular autophosphorylation, kinase activation and nuclear localization [57, 69, 81]. Mutation of Lys‐167 abrogates its kinase activity (Fig. 1). NLK is best known for its negative regulation of Wnt/β‐catenin signalling [82, 83]. It phosphorylates and binds to the transcription factor TCF/LEF1 and inhibits the β‐catenin‐TCF/LEF1 complex from interacting with DNA. Often, NLK functions downstream of the TGF‐beta activated kinase 1 (TAK1), which can be activated by TGF‐β, Wnt, and interleukin‐6 (IL‐6) [84, 85, 86, 87]. Furthermore, its ability to phosphorylate transcription factors and cofactors plays a role in various other signalling pathways, including Notch [88], p53 [89], NFκB [83], FOXO1 [90], STAT3 [84], and CCAAT/enhancer‐binding proteins (C/EBPs) [91].

Nemo‐like kinase is upregulated in several types of cancers (Fig. 4) and predicts poor prognosis [92]. It is considered as a tumour suppressor [93, 94, 95] or an oncogene [96, 97], respectively, depending on the context. For example, in human breast cancer cells, NLK has been reported to inhibit apoptosis by associating with heat shock protein [98], while in NSCLC, NLK has been shown to inhibit cancer progression and metastasis [99, 100].

## Atypical MAPKs in immune cell signalling

A fundamental question in oncology is how tumour cells escape destruction by cytotoxic immune cells. Two atypical MAPKs, ERK3 and NLK, are involved in the signal transduction in immune cells relevant in this context. ERK3 is expressed in thymocyte differentiation and plays a role in double‐positive (DP) thymocytes undergoing positive selection [101, 102]. This function of ERK3 is dependent on its kinase activity [101]. ERK3 was also shown to be involved in TCR‐induced activation of mature T cells [103]: While ERK3 is not expressed in resting T cells, TCR stimulation leads to ERK1/2‐dependent transcription of *ERK3* in both CD4^+^ and CD8^+^ T cells [103], and ERK3‐deficient T cells exhibit decreased proliferation and impaired cytokine production upon TCR stimulation. The molecular mechanism of action of ERK3 is not yet known, but may depend on MK5, which is induced simultaneously. Both CD4^+^ and CD8^+^ T cells are considered important anti‐tumour cells. It should be noted, however, that a differently engineered *ERK3* knockout mouse did not show abnormal T cell development, reduction of thymocyte numbers, or altered T cell selection [104]. Thus, it is possible that inhibiting ERK3 might be compensated by alternative pathways in immune signalling.

The TAK1‐NLK signalling pathway regulates the phosphorylation of FOXP3 in regulatory T (T_REG_) cells [105], which promote tumour progression by suppressing effective antitumour immunity. FOXP3 is a transcriptional regulator crucial for T_REG_ cell development and functional maintenance. Fleskens *et al*. reported that stimulation of TCR‐mediated signalling can induce the TAK1‐NLK pathway and sustain FOXP3 transcriptional activity by stabilizing the protein levels and preventing the association and degradation by STUB1 E3‐ubiquitin protein ligase, thereby maintaining T_REG_ cell suppressive function [94].

Nemo‐like kinase depletion promotes antiviral cytokine production and decreases viral replication. NLK phosphorylates MAVS (mitochondrial antiviral signalling protein), which is essential for antiviral immunity [106]. NLK's phosphorylation of MAVS at multiple sites on mitochondria or peroxisomes enables its degradation. The TRIM25‐RIG‐I‐MAVS axis has been shown to be important for eliciting antiviral immune response against HPV (human papilloma virus) and therefore HPV‐induced cervical or oropharyngeal cancers [107]. NLK inhibition could be an option for eliciting the required immune response against HPV infection, especially following cervical cancer screening programs that detect HPV‐positivity at CIN1 (cervical intraepithelial neoplasia, stage 1) or earlier stages.

### Bioinformatic analysis single‐cell RNA sequencing data of T cells

The roles of atypical MAPKs in immune cell signalling have been studied almost exclusively in mice. In order to ascertain the expression of atypical MAPKs in T cell activation and TCR signalling in humans, we performed bioinformatic analysis of single‐cell RNA sequencing data published by two studies downloaded from Gene Expression omnibus (GEO) under accession numbers GSE126030 [108] and GSE108989 [109]. Szabo *et al*. analysed the heterogeneity of T cells from multiple tissues (over 50 000 cells), including lungs, lymph nodes, bone marrow and blood, and their functional responses upon TCR stimulation using Human CD3/CD28 T Cell Activator (STEMCELL Technologies). Zhang *et al*. analysed the transcriptomes of 11 138 single T cells using STARTRAC (single T cell analysis by RNA sequencing and TCR tracking).

**Fig. 5:**
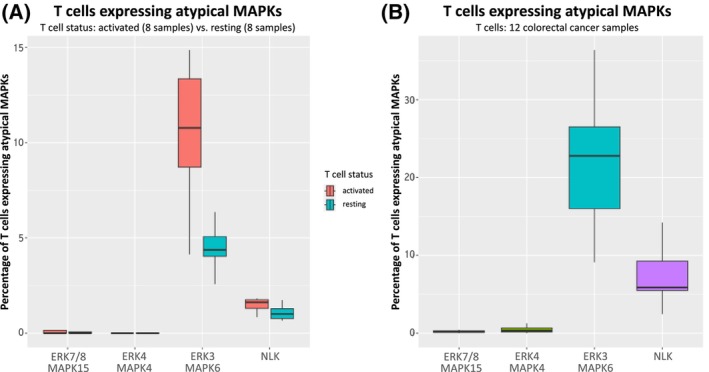
Expression of atypical MAPKs in human T cells (percentage of T cells). (A) Szabo *et al*. [108] used single‐cell RNA‐seq of over 50 000 resting and activated T cells from lungs, lymph nodes, bone marrow and blood, and analysed their functional responses following stimulation. They compared scRNA‐seq profiles of tumour‐associated T cells to their dataset and studied activated CD8^+^ compared to CD4^+^ T cell states within multiple tumour types. T cells were isolated, sorted, and sequenced from eight normal and eight stimulated samples of multiple tissues (lung, bone marrow and blood). For the shown plot, we extracted the atypical MAPKs associated with resting and activated cells. (B) We used the data from Zhang *et al*. [109] to show the expression of atypical MAPKs in the T cells of 12 colorectal cancer samples. The percentage of ERK3‐expressing T cells is higher than for other atypical MAPKs. Stimulated samples have a higher percentage of ERK3‐ and NLK‐expressing T cells than the respective unstimulated samples. A similar pattern is observed in cancer samples. In the context of immuno‐oncology and check point inhibitor therapy, the datasets such as these would further the understanding of immune response in cancer.

In our bioinformatic analysis, we calculated the percentage of resting and stimulated T cells (of 8 resting and 8 stimulated samples) that express atypical MAPKs, as well as the percentage of atypical MAPK‐expressing T cells isolated from 12 colorectal cancer samples (Fig. 5). We observed that the percentage of ERK3/MAPK6‐expressing T cells was significantly higher in stimulated than in resting T cells (Fig. 5A). In addition, in both resting and activated T cells the percentage of T cells expressing ERK3 was higher than of T cells expressing any other atypical MAPKs, suggesting that ERK3 plays the most important role in T cell function among the atypical MAPKs. Only NLK showed the same pattern as ERK3, though percentages of NLK‐expressing T cells are significantly lower than for ERK3 in both resting and stimulated T cells (Fig. 5A). Moreover, the percentage of T cells expressing ERK4 (MAPK4) and ERK7/8 (MAPK15) was low in both resting and stimulated T cells and any difference is negligible.

In T cells isolated from colorectal cancer, we have a similar pattern: The percentage of T cells expressing ERK3/MAPK6 and NLK is higher than the percentage of T cells expressing ERK4 (MAPK4) and ERK7/8 (MAPK15) (Fig. 5B). As the cancer samples are treatment naïve, it is likely that the observed expression is triggered by the cancer itself, and it remains to be investigated whether this is the cause or the effect of the T cell response or TCR activation. Our observation also aligns with the expression patterns of ERK3 and ERK4 in mice, and the known involvement of NLK in the activation of regulatory T cells [94].

## Summary and outlook

Although the studies are still limited, it is becoming increasingly clear that atypical MAPKs play crucial yet complex roles in cancer development, progression, and metastasis, and have great potential as drug targets. For the development of effective and efficient targeting strategies, however, further studies are needed to fully define their tissue‐ and cell type‐specific roles in health and disease. Also, discerning their kinase‐dependent and ‐independent functions in these contexts will be crucial. While conventional targeting strategies have focused primarily on inhibition of kinase activity by small molecule inhibitors, future targeting approaches will also include inhibitors of protein–protein interactions to target kinase‐independent functions while allowing kinase‐dependent functions to continue. In addition, proteolysis targeting chimeras (PROTACs) are promising tools that gain momentum in discovery and pre‐clinical phases [110]. Rather than acting as a classic inhibitor, PROTACs work by inducing selective proteolysis to permanently eliminate specific proteins. The approach has several pharmacological advantages and would target simultaneously kinase‐dependent and ‐independent activities of atypical MAPKs. Overall, atypical MAP kinases are a promising and emerging area of research and although they have been identified for some time now their physio‐ and patho‐physiological significance needs further studies. The role of these atypical MAPKs in the onco‐immune interface needs special attention as one may not want to dampen immune response(s) by targeting an essential kinase in an oncology setting. On the other hand further studies in this direction could also enable researchers to target atypical MAPKs in treating immune disorders. The large‐scale multiomic profiling of patients with cancer and immune disorders sheds some light into the deregulated kinome especially on atypical MAPKs which led to the development of first generation inhibitors. Kinase inhibitors have been very successful in treating cancers, especially when combined with classical chemo and/or immune therapeutic drugs. Targeting atypical MAPK perhaps in combinations may be the next step.

## Conflict of interest

The authors declare no conflict of interest.

## Author contributions

KD wrote the background information, and the sections on ERK3, ERK7/8 and NLK. PV made overall contributions to the manuscript. HW handled quality control of all sections and performed proof reading. HJS contributed conceptual ideas to the section “Atypical MAPKs in immune signalling”. KR conceived the entire review and contributed content and editing to all parts.

## References

[1] Cargnello M & Roux PP (2011) Activation and function of the MAPKs and their substrates, the MAPK‐activated protein kinases. Microbiol Mol Biol Rev 75, 50–83. 21372320 10.1128/MMBR.00031-10 PMC3063353

[2] Hadwiger JA , Aranda RG & Fatima S (2023) Atypical MAP kinases – new insights and directions from amoeba. J Cell Sci 136, jcs261447. 37850857 10.1242/jcs.261447 PMC10617611

[3] Bardwell L (2006) Mechanisms of MAPK signalling specificity. Biochem Soc Trans 34, 837–841. 17052210 10.1042/BST0340837 PMC3017501

[4] Martin‐Vega A & Cobb MH (2023) Navigating the ERK1/2 MAPK cascade. Biomolecules 13, 1555. 37892237 10.3390/biom13101555 PMC10605237

[5] Grewal S , Molina DM & Bardwell L (2006) Mitogen‐activated protein kinase (MAPK)‐docking sites in MAPK kinases function as tethers that are crucial for MAPK regulation in vivo. Cell Signal 18, 123–134. 15979847 10.1016/j.cellsig.2005.04.001 PMC3017502

[6] Peti W & Page R (2013) Molecular basis of MAP kinase regulation. Protein Sci 22, 1698–1710. 24115095 10.1002/pro.2374 PMC3843625

[7] Strniskova M , Barancik M & Ravingerova T (2002) Mitogen‐activated protein kinases and their role in regulation of cellular processes. Gen Physiol Biophys 21, 231–255. 12537349

[8] Ng GYQ , Loh ZW , Fann DY , Mallilankaraman K , Arumugam TV & Hande MP (2024) Role of mitogen‐activated protein (MAP) kinase pathways in metabolic diseases. Genome Integr 15, e20230003. 38770527 10.14293/genint.14.1.004 PMC11102075

[9] Lucas RM , Luo L & Stow JL (2022) ERK1/2 in immune signalling. Biochem Soc Trans 50, 1341–1352. 36281999 10.1042/BST20220271 PMC9704528

[10] Braicu C , Buse M , Busuioc C , Drula R , Gulei D , Raduly L , Rusu A , Irimie A , Atanasov AG , Slaby O *et al*. (2019) A comprehensive review on MAPK: a promising therapeutic target in cancer. Cancers (Basel) 11, 1618. 31652660 10.3390/cancers11101618 PMC6827047

[11] Aberg E , Torgersen KM , Johansen B , Keyse SM , Perander M & Seternes OM (2009) Docking of PRAK/MK5 to the atypical MAPKs ERK3 and ERK4 defines a novel MAPK interaction motif. J Biol Chem 284, 19392–19401. 19473979 10.1074/jbc.M109.023283 PMC2740564

[12] An HJ , Lee CJ , Lee GE , Choi Y , Jeung D , Chen W , Lee HS , Kang HC , Lee JY , Kim DJ *et al*. (2022) FBXW7‐mediated ERK3 degradation regulates the proliferation of lung cancer cells. Exp Mol Med 54, 35–46. 35022544 10.1038/s12276-021-00721-9 PMC8813941

[13] Togi S , Ikeda O , Kamitani S , Nakasuji M , Sekine Y , Muromoto R , Nanbo A , Oritani K , Kawai T , Akira S *et al*. (2011) Zipper‐interacting protein kinase (ZIPK) modulates canonical Wnt/beta‐catenin signaling through interaction with Nemo‐like kinase and T‐cell factor 4 (NLK/TCF4). J Biol Chem 286, 19170–19177. 21454679 10.1074/jbc.M110.189829 PMC3099730

[14] Aberg E , Perander M , Johansen B , Julien C , Meloche S , Keyse SM & Seternes OM (2006) Regulation of MAPK‐activated protein kinase 5 activity and subcellular localization by the atypical MAPK ERK4/MAPK4. J Biol Chem 281, 35499–35510. 16971392 10.1074/jbc.M606225200

[15] Kant S , Schumacher S , Singh MK , Kispert A , Kotlyarov A & Gaestel M (2006) Characterization of the atypical MAPK ERK4 and its activation of the MAPK‐activated protein kinase MK5. J Biol Chem 281, 35511–35519. 16973613 10.1074/jbc.M606693200

[16] Seternes OM , Mikalsen T , Johansen B , Michaelsen E , Armstrong CG , Morrice NA , Turgeon B , Meloche S , Moens U & Keyse SM (2004) Activation of MK5/PRAK by the atypical MAP kinase ERK3 defines a novel signal transduction pathway. EMBO J 23, 4780–4791. 15577943 10.1038/sj.emboj.7600489 PMC535098

[17] Anhe GF , Torrao AS , Nogueira TC , Caperuto LC , Amaral ME , Medina MC , Azevedo‐Martins AK , Carpinelli AR , Carvalho CR , Curi R *et al*. (2006) ERK3 associates with MAP2 and is involved in glucose‐induced insulin secretion. Mol Cell Endocrinol 251, 33–41. 16597486 10.1016/j.mce.2006.02.012

[18] Kassouf T & Sumara G (2020) Impact of conventional and atypical MAPKs on the development of metabolic diseases. Biomolecules 10, 1256. 32872540 10.3390/biom10091256 PMC7563211

[19] Bian K , Muppani NR , Elkhadragy L , Wang W , Zhang C , Chen T , Jung S , Seternes OM & Long W (2016) ERK3 regulates TDP2‐mediated DNA damage response and chemoresistance in lung cancer cells. Oncotarget 7, 6665–6675. 26701725 10.18632/oncotarget.6682 PMC4872741

[20] Long W , Foulds CE , Qin J , Liu J , Ding C , Lonard DM , Solis LM , Wistuba II , Qin J , Tsai SY *et al*. (2012) ERK3 signals through SRC‐3 coactivator to promote human lung cancer cell invasion. J Clin Invest 122, 1869–1880. 22505454 10.1172/JCI61492 PMC3336992

[21] Brand F , Schumacher S , Kant S , Menon MB , Simon R , Turgeon B , Britsch S , Meloche S , Gaestel M & Kotlyarov A (2012) The extracellular signal‐regulated kinase 3 (mitogen‐activated protein kinase 6 [MAPK6])‐MAPK‐activated protein kinase 5 signaling complex regulates septin function and dendrite morphology. Mol Cell Biol 32, 2467–2478. 22508986 10.1128/MCB.06633-11 PMC3434500

[22] Javary J , Goupil E , Soulez M , Kanshin E , Bouchard A , Seternes OM , Thibault P , Labbé JC & Meloche S (2022) Phosphoproteomic analysis identifies supervillin as an ERK3 substrate regulating cytokinesis and cell ploidy. J Cell Physiol 239, e30938. 36576983 10.1002/jcp.30938

[23] Myers AK , Morel M , Gee SH , Hoffmann KA & Long W (2023) ERK3 and DGKζ interact to modulate cell motility in lung cancer cells. Front Cell Dev Biol 11, 1192221. 37287450 10.3389/fcell.2023.1192221 PMC10242005

[24] Bogucka‐Janczi K , Harms G , Coissieux MM , Bentires‐Alj M , Thiede B & Rajalingam K (2023) ERK3/MAPK6 dictates CDC42/RAC1 activity and ARP2/3‐dependent actin polymerization. Elife 12, e85167. 37057894 10.7554/eLife.85167 PMC10191626

[25] Cai Q , Zhou W , Wang W , Dong B , Han D , Shen T , Creighton CJ , Moore DD & Yang F (2021) MAPK6‐AKT signaling promotes tumor growth and resistance to mTOR kinase blockade. Sci Adv 7, eabi6439. 34767444 10.1126/sciadv.abi6439 PMC8589317

[26] Perander M , Keyse SM & Seternes OM (2016) New insights into the activation, interaction partners and possible functions of MK5/PRAK. Front Biosci (Landmark Ed) 21, 374–384. 26709779 10.2741/4394

[27] Coulombe P , Rodier G , Bonneil E , Thibault P & Meloche S (2004) N‐terminal ubiquitination of extracellular signal‐regulated kinase 3 and p21 directs their degradation by the proteasome. Mol Cell Biol 24, 6140–6150. 15226418 10.1128/MCB.24.14.6140-6150.2004 PMC434260

[28] Coulombe P , Rodier G , Pelletier S , Pellerin J & Meloche S (2003) Rapid turnover of extracellular signal‐regulated kinase 3 by the ubiquitin‐proteasome pathway defines a novel paradigm of mitogen‐activated protein kinase regulation during cellular differentiation. Mol Cell Biol 23, 4542–4558. 12808096 10.1128/MCB.23.13.4542-4558.2003 PMC164847

[29] Mathien S , Déléris P , Soulez M , Voisin L & Meloche S (2017) Deubiquitinating enzyme USP20 regulates extracellular signal‐regulated kinase 3 stability and biological activity. Mol Cell Biol 37, e00432‐16. 28167606 10.1128/MCB.00432-16 PMC5394282

[30] Deleris P , Trost M , Topisirovic I , Tanguay PL , Borden KL , Thibault P & Meloche S (2011) Activation loop phosphorylation of ERK3/ERK4 by group I p21‐activated kinases (PAKs) defines a novel PAK‐ERK3/4‐MAPK‐activated protein kinase 5 signaling pathway. J Biol Chem 286, 6470–6478. 21177870 10.1074/jbc.M110.181529 PMC3057823

[31] Bogucka K , Marini F , Rosigkeit S , Schloeder J , Jonuleit H , David K , Schlackow M & Rajalingam K (2021) ERK3/MAPK6 is required for KRAS‐mediated NSCLC tumorigenesis. Cancer Gene Ther 28, 359–374. 33070159 10.1038/s41417-020-00245-w

[32] Julien C , Coulombe P & Meloche S (2003) Nuclear export of ERK3 by a CRM1‐dependent mechanism regulates its inhibitory action on cell cycle progression. J Biol Chem 278, 42615–42624. 12915405 10.1074/jbc.M302724200

[33] Alsaran H , Elkhadragy L , Shakya A & Long W (2017) L290P/V mutations increase ERK3's cytoplasmic localization and migration/invasion‐promoting capability in cancer cells. Sci Rep 7, 14979. 29101390 10.1038/s41598-017-15135-9 PMC5670241

[34] Elkhadragy L , Alsaran H , Morel M & Long W (2018) Activation loop phosphorylation of ERK3 is important for its kinase activity and ability to promote lung cancer cell invasiveness. J Biol Chem 293, 16193–16205. 30166347 10.1074/jbc.RA118.003699 PMC6200930

[35] Al‐Mahdi R , Babteen N , Thillai K , Holt M , Johansen B , Wetting HL , Seternes OM & Wells CM (2015) A novel role for atypical MAPK kinase ERK3 in regulating breast cancer cell morphology and migration. Cell Adh Migr 9, 483–494. 26588708 10.1080/19336918.2015.1112485 PMC4955959

[36] Bogucka K , Pompaiah M , Marini F , Binder H , Harms G , Kaulich M , Klein M , Michel C , Radsak MP , Rosigkeit S *et al*. (2020) ERK3/MAPK6 controls IL‐8 production and chemotaxis. Elife 9, e52511. 32314963 10.7554/eLife.52511 PMC7192585

[37] Soulez M , Tanguay PL , Do F , Dort J , Crist C , Kotlyarov A , Gaestel M , Ferron M , Dumont NA & Meloche S (2022) ERK3‐MK5 signaling regulates myogenic differentiation and muscle regeneration by promoting FoxO3 degradation. J Cell Physiol 237, 2271–2287. 35141958 10.1002/jcp.30695

[38] El‐Merahbi R , Viera JT , Valdes AL , Kolczynska K , Reuter S , Loffler MC , Erk M , Ade CP , Karwen T , Mayer AE *et al*. (2020) The adrenergic‐induced ERK3 pathway drives lipolysis and suppresses energy dissipation. Genes Dev 34, 495–510. 32139423 10.1101/gad.333617.119 PMC7111262

[39] Bhattacharjee A , Richards WG , Staunton J , Li C , Monti S , Vasa P , Ladd C , Beheshti J , Bueno R , Gillette M *et al*. (2001) Classification of human lung carcinomas by mRNA expression profiling reveals distinct adenocarcinoma subclasses. Proc Natl Acad Sci USA 98, 13790–13795. 11707567 10.1073/pnas.191502998 PMC61120

[40] Evtimova V , Schwirzke M , Tarbe N , Burtscher H , Jarsch M , Kaul S & Weidle UH (2001) Identification of breast cancer metastasis‐associated genes by chip technology. Anticancer Res 21, 3799–3806. 11911250

[41] Liang B , Wang S , Zhu XG , Yu YX , Cui ZR & Yu YZ (2005) Increased expression of mitogen‐activated protein kinase and its upstream regulating signal in human gastric cancer. World J Gastroenterol 11, 623–628. 15655810 10.3748/wjg.v11.i5.623 PMC4250727

[42] Nambiar S , Mirmohammadsadegh A , Doroudi R , Gustrau A , Marini A , Roeder G , Ruzicka T & Hengge UR (2005) Signaling networks in cutaneous melanoma metastasis identified by complementary DNA microarrays. Arch Dermatol 141, 165–173. 15724012 10.1001/archderm.141.2.165

[43] Chen M , Myers AK , Markey MP & Long W (2019) The atypical MAPK ERK3 potently suppresses melanoma cell growth and invasiveness. J Cell Physiol 234, 13220–13232. 30569573 10.1002/jcp.27994 PMC8378996

[44] Elkhadragy L , Alsaran H & Long W (2020) The C‐terminus tail regulates ERK3 kinase activity and its ability in promoting cancer cell migration and invasion. Int J Mol Sci 21, 4044. 32516969 10.3390/ijms21114044 PMC7312006

[45] Gradler U , Busch M , Leuthner B , Raba M , Burgdorf L , Lehmann M , Linde N & Esdar C (2020) Biochemical, cellular and structural characterization of novel and selective ERK3 inhibitors. Bioorg Med Chem Lett 30, 127551. 32927028 10.1016/j.bmcl.2020.127551

[46] Riese MJ , Moon EK , Johnson BD & Albelda SM (2016) Diacylglycerol kinases (DGKs): novel targets for improving T cell activity in cancer. Front Cell Dev Biol 4, 108. 27800476 10.3389/fcell.2016.00108 PMC5065962

[47] Perander M , Aberg E , Johansen B , Dreyer B , Guldvik IJ , Outzen H , Keyse SM & Seternes OM (2008) The Ser(186) phospho‐acceptor site within ERK4 is essential for its ability to interact with and activate PRAK/MK5. Biochem J 411, 613–622. 18248330 10.1042/BJ20071369

[48] Deleris P , Rousseau J , Coulombe P , Rodier G , Tanguay PL & Meloche S (2008) Activation loop phosphorylation of the atypical MAP kinases ERK3 and ERK4 is required for binding, activation and cytoplasmic relocalization of MK5. J Cell Physiol 217, 778–788. 18720373 10.1002/jcp.21560

[49] Perander M , Al‐Mahdi R , Jensen TC , Nunn JA , Kildalsen H , Johansen B , Gabrielsen M , Keyse SM & Seternes OM (2017) Regulation of atypical MAP kinases ERK3 and ERK4 by the phosphatase DUSP2. Sci Rep 7, 43471. 28252035 10.1038/srep43471 PMC5333157

[50] Rousseau J , Klinger S , Rachalski A , Turgeon B , Déléris P , Vigneault E , Poirier‐Héon JF , Davoli MA , Mechawar N , el Mestikawy S *et al*. (2010) Targeted inactivation of Mapk4 in mice reveals specific nonredundant functions of Erk3/Erk4 subfamily mitogen‐activated protein kinases. Mol Cell Biol 30, 5752–5763. 20956558 10.1128/MCB.01147-10 PMC3004264

[51] Klinger S , Turgeon B , Lévesque K , Wood GA , Aagaard‐Tillery KM & Meloche S (2009) Loss of Erk3 function in mice leads to intrauterine growth restriction, pulmonary immaturity, and neonatal lethality. Proc Natl Acad Sci USA 106, 16710–16715. 19805361 10.1073/pnas.0900919106 PMC2757836

[52] Gillespie M , Jassal B , Stephan R , Milacic M , Rothfels K , Senff‐Ribeiro A , Griss J , Sevilla C , Matthews L , Gong C *et al*. (2022) The reactome pathway knowledgebase 2022. Nucleic Acids Res 50, D687–D692. 34788843 10.1093/nar/gkab1028 PMC8689983

[53] Szklarczyk D , Franceschini A , Wyder S , Forslund K , Heller D , Huerta‐Cepas J , Simonovic M , Roth A , Santos A , Tsafou KP *et al*. (2015) STRING v10: protein‐protein interaction networks, integrated over the tree of life. Nucleic Acids Res 43, D447–D452. 25352553 10.1093/nar/gku1003 PMC4383874

[54] Hermjakob H , Montecchi‐Palazzi L , Lewington C , Mudali S , Kerrien S , Orchard S , Vingron M , Roechert B , Roepstorff P , Valencia A *et al*. (2004) IntAct: an open source molecular interaction database. Nucleic Acids Res 32, D452–D455. 14681455 10.1093/nar/gkh052 PMC308786

[55] AACR Project GENIE Consortium (2017) AACR project GENIE: powering precision medicine through an international consortium. Cancer Discov 7, 818–831. 28572459 10.1158/2159-8290.CD-17-0151 PMC5611790

[56] Abe MK , Saelzler MP , Espinosa R 3rd , Kahle KT , Hershenson MB , Le Beau MM & Rosner MR (2002) ERK8, a new member of the mitogen‐activated protein kinase family. J Biol Chem 277, 16733–16743. 11875070 10.1074/jbc.M112483200

[57] Abe MK , Kuo WL , Hershenson MB & Rosner MR (1999) Extracellular signal‐regulated kinase 7 (ERK7), a novel ERK with a C‐terminal domain that regulates its activity, its cellular localization, and cell growth. Mol Cell Biol 19, 1301–1312. 9891064 10.1128/mcb.19.2.1301 PMC116059

[58] Tweedie S , Braschi B , Gray K , Jones TEM , Seal RL , Yates B & Bruford EA (2021) Genenames.org: the HGNC and VGNC resources in 2021. Nucleic Acids Res 49, D939–D946. 33152070 10.1093/nar/gkaa980 PMC7779007

[59] Lau ATY & Xu YM (2018) Regulation of human mitogen‐activated protein kinase 15 (extracellular signal‐regulated kinase 7/8) and its functions: a recent update. J Cell Physiol 234, 75–88. 30070699 10.1002/jcp.27053

[60] Klevernic IV , Stafford MJ , Morrice N , Peggie M , Morton S & Cohen P (2006) Characterization of the reversible phosphorylation and activation of ERK8. Biochem J 394, 365–373. 16336213 10.1042/BJ20051288 PMC1386035

[61] Abe MK , Kahle KT , Saelzler MP , Orth K , Dixon JE & Rosner MR (2001) ERK7 is an autoactivated member of the MAPK family. J Biol Chem 276, 21272–21279. 11287416 10.1074/jbc.M100026200

[62] Kuo WL , Duke CJ , Abe MK , Kaplan EL , Gomes S & Rosner MR (2004) ERK7 expression and kinase activity is regulated by the ubiquitin‐proteosome pathway. J Biol Chem 279, 23073–23081. 15033983 10.1074/jbc.M313696200

[63] Erster O , Seger R & Liscovitch M (2010) Ligand interaction scan (LIScan) in the study of ERK8. Biochem Biophys Res Commun 399, 37–41. 20638370 10.1016/j.bbrc.2010.07.029

[64] Colecchia D , Strambi A , Sanzone S , Iavarone C , Rossi M , Dall'Armi C , Piccioni F , Verrotti di Pianella A & Chiariello M (2012) MAPK15/ERK8 stimulates autophagy by interacting with LC3 and GABARAP proteins. Autophagy 8, 1724–1740. 22948227 10.4161/auto.21857 PMC3541284

[65] Chia J , Tham KM , Gill DJ , Bard‐Chapeau EA & Bard FA (2014) ERK8 is a negative regulator of O‐GalNAc glycosylation and cell migration. Elife 3, e01828. 24618899 10.7554/eLife.01828 PMC3945522

[66] Groehler AL & Lannigan DA (2010) A chromatin‐bound kinase, ERK8, protects genomic integrity by inhibiting HDM2‐mediated degradation of the DNA clamp PCNA. J Cell Biol 190, 575–586. 20733054 10.1083/jcb.201002124 PMC2928013

[67] Rossi M , Colecchia D , Iavarone C , Strambi A , Piccioni F , Verrotti di Pianella A & Chiariello M (2011) Extracellular signal‐regulated kinase 8 (ERK8) controls estrogen‐related receptor alpha (ERRalpha) cellular localization and inhibits its transcriptional activity. J Biol Chem 286, 8507–8522. 21190936 10.1074/jbc.M110.179523 PMC3048734

[68] Wu DD , Lau ATY , Yu FY , Cai NL , Dai LJ , Ok Kim M , Jin DY & Xu YM (2017) Extracellular signal‐regulated kinase 8‐mediated NF‐kappaB activation increases sensitivity of human lung cancer cells to arsenic trioxide. Oncotarget 8, 49144–49155. 28467781 10.18632/oncotarget.17100 PMC5564756

[69] Henrich LM , Smith JA , Kitt D , Errington TM , Nguyen B , Traish AM & Lannigan DA (2003) Extracellular signal‐regulated kinase 7, a regulator of hormone‐dependent estrogen receptor destruction. Mol Cell Biol 23, 5979–5988. 12917323 10.1128/MCB.23.17.5979-5988.2003 PMC180983

[70] Saelzler MP , Spackman CC , Liu Y , Martinez LC , Harris JP & Abe MK (2006) ERK8 down‐regulates transactivation of the glucocorticoid receptor through Hic‐5. J Biol Chem 281, 16821–16832. 16624805 10.1074/jbc.M512418200

[71] Brennan A , Leech JT , Kad NM & Mason JM (2020) Selective antagonism of cJun for cancer therapy. J Exp Clin Cancer Res 39, 184. 32917236 10.1186/s13046-020-01686-9 PMC7488417

[72] Meng Q & Xia Y (2011) c‐Jun, at the crossroad of the signaling network. Protein Cell 2, 889–898. 22180088 10.1007/s13238-011-1113-3 PMC4875184

[73] Xu YM , Zhu F , Cho YY , Carper A , Peng C , Zheng D , Yao K , Lau ATY , Zykova TA , Kim HG *et al*. (2010) Extracellular signal‐regulated kinase 8‐mediated c‐Jun phosphorylation increases tumorigenesis of human colon cancer. Cancer Res 70, 3218–3227. 20395206 10.1158/0008-5472.CAN-09-4306

[74] Su Z , Yang B , Zeng Z , Zhu S , Wang C , Lei S , Jiang Y & Lin L (2020) Metastasis‐associated gene MAPK15 promotes the migration and invasion of osteosarcoma cells via the c‐Jun/MMPs pathway. Oncol Lett 20, 99–112. 10.3892/ol.2020.11544 PMC7285714 32565938

[75] Jin DH , Lee J , Kim KM , Kim S , Kim DH & Park J (2015) Overexpression of MAPK15 in gastric cancer is associated with copy number gain and contributes to the stability of c‐Jun. Oncotarget 6, 20190–20203. 26035356 10.18632/oncotarget.4171 PMC4652997

[76] Colecchia D , Rossi M , Sasdelli F , Sanzone S , Strambi A & Chiariello M (2015) MAPK15 mediates BCR‐ABL1‐induced autophagy and regulates oncogene‐dependent cell proliferation and tumor formation. Autophagy 11, 1790–1802. 26291129 10.1080/15548627.2015.1084454 PMC4824572

[77] Helgason GV , Karvela M & Holyoake TL (2011) Kill one bird with two stones: potential efficacy of BCR‐ABL and autophagy inhibition in CML. Blood 118, 2035–2043. 21693757 10.1182/blood-2011-01-330621

[78] Kazatskaya A , Kuhns S , Lambacher NJ , Kennedy JE , Brear AG , McManus GJ , Sengupta P & Blacque OE (2017) Primary cilium formation and ciliary protein trafficking is regulated by the atypical MAP kinase MAPK15 in *Caenorhabditis elegans* and human cells. Genetics 207, 1423–1440. 29021280 10.1534/genetics.117.300383 PMC5714457

[79] Miyatake K , Kusakabe M , Takahashi C & Nishida E (2015) ERK7 regulates ciliogenesis by phosphorylating the actin regulator CapZIP in cooperation with dishevelled. Nat Commun 6, 6666. 25823377 10.1038/ncomms7666

[80] Pietrobono S , Franci L , Imperatore F , Zanini C , Stecca B & Chiariello M (2021) MAPK15 controls hedgehog signaling in medulloblastoma cells by regulating primary ciliogenesis. Cancers (Basel) 13, 4903. 34638386 10.3390/cancers13194903 PMC8508543

[81] Ishitani S , Inaba K , Matsumoto K & Ishitani T (2011) Homodimerization of Nemo‐like kinase is essential for activation and nuclear localization. Mol Biol Cell 22, 266–277. 21118996 10.1091/mbc.E10-07-0605 PMC3020921

[82] Ishitani T , Ninomiya‐Tsuji J , Nagai S , Nishita M , Meneghini M , Barker N , Waterman M , Bowerman B , Clevers H , Shibuya H *et al*. (1999) The TAK1‐NLK‐MAPK‐related pathway antagonizes signalling between beta‐catenin and transcription factor TCF. Nature 399, 798–802. 10391247 10.1038/21674

[83] Yasuda J , Yokoo H , Yamada T , Kitabayashi I , Sekiya T & Ichikawa H (2004) Nemo‐like kinase suppresses a wide range of transcription factors, including nuclear factor‐kappaB. Cancer Sci 95, 52–57. 14720327 10.1111/j.1349-7006.2004.tb03170.x PMC11158368

[84] Ohkawara B , Shirakabe K , Hyodo‐Miura J , Matsuo R , Ueno N , Matsumoto K & Shibuya H (2004) Role of the TAK1‐NLK‐STAT3 pathway in TGF‐beta‐mediated mesoderm induction. Genes Dev 18, 381–386. 15004007 10.1101/gad.1166904 PMC359392

[85] Ishitani T , Ninomiya‐Tsuji J & Matsumoto K (2003) Regulation of lymphoid enhancer factor 1/T‐cell factor by mitogen‐activated protein kinase‐related Nemo‐like kinase‐dependent phosphorylation in Wnt/beta‐catenin signaling. Mol Cell Biol 23, 1379–1389. 12556497 10.1128/MCB.23.4.1379-1389.2003 PMC141159

[86] Meneghini MD , Ishitani T , Carter JC , Hisamoto N , Ninomiya‐Tsuji J , Thorpe CJ , Hamill DR , Matsumoto K & Bowerman B (1999) MAP kinase and Wnt pathways converge to downregulate an HMG‐domain repressor in *Caenorhabditis elegans* . Nature 399, 793–797. 10391246 10.1038/21666

[87] Kanei‐Ishii C , Ninomiya‐Tsuji J , Tanikawa J , Nomura T , Ishitani T , Kishida S , Kokura K , Kurahashi T , Ichikawa‐Iwata E , Kim Y *et al*. (2004) Wnt‐1 signal induces phosphorylation and degradation of c‐Myb protein via TAK1, HIPK2, and NLK. Genes Dev 18, 816–829. 15082531 10.1101/gad.1170604 PMC387421

[88] Ishitani T , Hirao T , Suzuki M , Isoda M , Ishitani S , Harigaya K , Kitagawa M , Matsumoto K & Itoh M (2010) Nemo‐like kinase suppresses notch signalling by interfering with formation of the notch active transcriptional complex. Nat Cell Biol 12, 278–285. 20118921 10.1038/ncb2028

[89] Zhang HH , Li SZ , Zhang ZY , Hu XM , Hou PN , Gao L , Du RL & Zhang XD (2014) Nemo‐like kinase is critical for p53 stabilization and function in response to DNA damage. Cell Death Differ 21, 1656–1663. 24926618 10.1038/cdd.2014.78 PMC4158690

[90] Kim S , Kim Y , Lee J & Chung J (2010) Regulation of FOXO1 by TAK1‐Nemo‐like kinase pathway. J Biol Chem 285, 8122–8129. 20061393 10.1074/jbc.M110.101824 PMC2832963

[91] Zhang ZY , Li SZ , Zhang HH , Wu QR , Gong J , Liang T , Gao L , Xing NN , Liu WB , Du RL *et al*. (2015) Stabilization of ATF5 by TAK1‐Nemo‐like kinase critically regulates the interleukin‐1beta‐stimulated C/EBP signaling pathway. Mol Cell Biol 35, 778–788. 25512613 10.1128/MCB.01228-14 PMC4323494

[92] Huang Y , Yang Y , He Y & Li J (2015) The emerging role of Nemo‐like kinase (NLK) in the regulation of cancers. Tumour Biol 36, 9147–9152. 26427665 10.1007/s13277-015-4159-7

[93] Wang J , Yang ZH , Chen H , Li HH , Chen LY , Zhu Z , Zou Y , Ding CC , Yang J & He ZW (2016) Nemo‐like kinase as a negative regulator of nuclear receptor Nurr1 gene transcription in prostate cancer. BMC Cancer 16, 257. 27036119 10.1186/s12885-016-2291-4 PMC4815267

[94] Huang Y , Jiang Y , Lu W & Zhang Y (2013) Nemo‐like kinase associated with proliferation and apoptosis by c‐Myb degradation in breast cancer. PLoS One 8, e69148. 23935942 10.1371/journal.pone.0069148 PMC3720543

[95] Emami KH , Brown LG , Pitts TE , Sun X , Vessella RL & Corey E (2009) Nemo‐like kinase induces apoptosis and inhibits androgen receptor signaling in prostate cancer cells. Prostate 69, 1481–1492. 19514049 10.1002/pros.20998 PMC2908180

[96] Li SZ , Zeng F , Li J , Shu QP , Zhang HH , Xu J , Ren JW , Zhang XD , Song XM & Du RL (2018) Nemo‐like kinase (NLK) primes colorectal cancer progression by releasing the E2F1 complex from HDAC1. Cancer Lett 431, 43–53. 29803790 10.1016/j.canlet.2018.05.032

[97] Suwei D , Liang Z , Zhimin L , Ruilei L , Yingying Z , Zhen L , Chunlei G , Zhangchao L , Yuanbo X , Jinyan Y *et al*. (2015) NLK functions to maintain proliferation and stemness of NSCLC and is a target of metformin. J Hematol Oncol 8, 120. 26503334 10.1186/s13045-015-0203-8 PMC4620602

[98] Shaw‐Hallgren G , Chmielarska Masoumi K , Zarrizi R , Hellman U , Karlsson P , Helou K & Massoumi R (2014) Association of nuclear‐localized Nemo‐like kinase with heat‐shock protein 27 inhibits apoptosis in human breast cancer cells. PLoS One 9, e96506. 24816797 10.1371/journal.pone.0096506 PMC4015990

[99] Shi C , Xu L , Tang Z , Zhang W , Wei Y , Ni J , Zhang S & Feng J (2019) Knockdown of Nemo‐like kinase promotes metastasis in nonsmallcell lung cancer. Oncol Rep 42, 1090–1100. 31322229 10.3892/or.2019.7226 PMC6667924

[100] Lv L , Wan C , Chen B , Li M , Liu Y , Ni T , Yang Y , Liu Y , Cong X , Mao G *et al*. (2014) Nemo‐like kinase (NLK) inhibits the progression of NSCLC via negatively modulating WNT signaling pathway. J Cell Biochem 115, 81–92. 23904219 10.1002/jcb.24635

[101] Marquis M , Daudelin JF , Boulet S , Sirois J , Crain K , Mathien S , Turgeon B , Rousseau J , Meloche S & Labrecque N (2014) The catalytic activity of the mitogen‐activated protein kinase extracellular signal‐regulated kinase 3 is required to sustain CD4+ CD8+ thymocyte survival. Mol Cell Biol 34, 3374–3387. 25002529 10.1128/MCB.01701-13 PMC4135614

[102] Sirois J , Daudelin JF , Boulet S , Marquis M , Meloche S & Labrecque N (2015) The atypical MAPK ERK3 controls positive selection of thymocytes. Immunology 145, 161–169. 25521218 10.1111/imm.12433 PMC4405333

[103] Marquis M , Boulet S , Mathien S , Rousseau J , Thébault P , Daudelin JF , Rooney J , Turgeon B , Beauchamp C , Meloche S *et al*. (2014) The non‐classical MAP kinase ERK3 controls T cell activation. PLoS One 9, e86681. 24475167 10.1371/journal.pone.0086681 PMC3903551

[104] Ronkina N , Schuster‐Gossler K , Hansmann F , Kunze‐Schumacher H , Sandrock I , Yakovleva T , Lafera J , Baumgärtner W , Krueger A , Prinz I *et al*. (2019) Germ line deletion reveals a nonessential role of atypical mitogen‐activated protein kinase 6/extracellular signal‐regulated kinase 3. Mol Cell Biol 39, e00516‐18. 30642948 10.1128/MCB.00516-18 PMC6399663

[105] Fleskens V , Minutti CM , Wu X , Wei P , Pals C , McCrae J , Hemmers S , Groenewold V , Vos HJ , Rudensky A *et al*. (2019) Nemo‐like kinase drives Foxp3 stability and is critical for maintenance of immune tolerance by regulatory T cells. Cell Rep 26, 3600–3612.e6. 30917315 10.1016/j.celrep.2019.02.087 PMC6444001

[106] Li S‐Z , Shu Q‐P , Song Y , Zhang H‐H , Liu Y , Jin B‐X , Liuyu TZ , Li C , Huang XC , Du RL *et al*. (2019) Phosphorylation of MAVS/VISA by Nemo‐like kinase (NLK) for degradation regulates the antiviral innate immune response. Nat Commun 10, 3233. 31324787 10.1038/s41467-019-11258-x PMC6642205

[107] Chiang C , Pauli EK , Biryukov J , Feister KF , Meng M , White EA , Münger K , Howley PM , Meyers C & Gack MU (2018) The human papillomavirus E6 oncoprotein targets USP15 and TRIM25 to suppress RIG‐I‐mediated innate immune signaling. J Virol 92, e01737‐17. 29263274 10.1128/JVI.01737-17 PMC5827370

[108] Szabo PA , Levitin HM , Miron M , Snyder ME , Senda T , Yuan J , Cheng YL , Bush EC , Dogra P , Thapa P *et al*. (2019) Single‐cell transcriptomics of human T cells reveals tissue and activation signatures in health and disease. Nat Commun 10, 4706. 31624246 10.1038/s41467-019-12464-3 PMC6797728

[109] Zhang Y , Zheng L , Zhang L , Hu X , Ren X & Zhang Z (2019) Deep single‐cell RNA sequencing data of individual T cells from treatment‐naïve colorectal cancer patients. Sci Data 6, 131. 31341169 10.1038/s41597-019-0131-5 PMC6656756

[110] Li X , Pu W , Zheng Q , Ai M , Chen S & Peng Y (2022) Proteolysis‐targeting chimeras (PROTACs) in cancer therapy. Mol Cancer 21, 99. 35410300 10.1186/s12943-021-01434-3 PMC8996410

[111] Abramson J , Adler J , Dunger J , Evans R , Green T , Pritzel A , Ronneberger O , Willmore L , Ballard AJ , Bambrick J *et al*. (2024) Accurate structure prediction of biomolecular interactions with AlphaFold 3. Nature 630, 493–500. 38718835 10.1038/s41586-024-07487-w PMC11168924

[112] Chandrashekar DS , Karthikeyan SK , Korla PK , Patel H , Shovon AR , Athar M , Netto GJ , Qin ZS , Kumar S , Manne U *et al*. (2022) UALCAN: an update to the integrated cancer data analysis platform. Neoplasia 25, 18–27. 35078134 10.1016/j.neo.2022.01.001 PMC8788199

[113] Heath AP , Ferretti V , Agrawal S , An M , Angelakos JC , Arya R , Bajari R , Baqar B , Barnowski JHB , Burt J *et al*. (2021) The NCI genomic data commons. Nat Genet 53, 257–262. 33619384 10.1038/s41588-021-00791-5

